# Heavy Chain-Hyaluronan/Pentraxin 3 from Amniotic Membrane Suppresses Inflammation and Scarring in Murine Lacrimal Gland and Conjunctiva of Chronic Graft-versus-Host Disease

**DOI:** 10.1038/srep42195

**Published:** 2017-02-06

**Authors:** Yoko Ogawa, Hua He, Shin Mukai, Toshihiro Imada, Shigeru Nakamura, Chen-Wei Su, Megha Mahabole, Scheffer C. G. Tseng, Kazuo Tsubota

**Affiliations:** 1 Department of Ophthalmology, Keio University School of Medicine, 35 Shinanomachi Shinjuku-ku, Tokyo, 160-8582, Japan; 2 TissueTech, Inc., Miami, FL 33173, USA

## Abstract

Chronic graft-versus-host disease (cGVHD) is a major complication of hematopoietic stem cell transplantation. Dry eye disease is the prominent ocular sequel of cGVHD and is caused by excessive inflammation and fibrosis in the lacrimal glands. Heavy chain-Hyaluronan/Pentraxin 3 (HC-HA/PTX3) is a complex purified from human amniotic membrane (AM) and known to exert anti-inflammatory and anti-scarring actions. In this study, we utilized a mouse model of cGVHD to examine whether HC-HA/PTX3 could attenuate dry eye disease elicited by cGVHD. Our results indicated that subconjunctival and subcutaneous injection of HC-HA/PTX3 preserved tear secretion and conjunctival goblet cell density and mitigated inflammation and scarring of the conjunctiva. Such therapeutic benefits were associated with suppression of scarring and infiltration of inflammatory/immune cells in the lacrimal glands. Furthermore, HC-HA/PTX3 significantly reduced the extent of infiltration of CD45^+^ CD4^+^ IL-17^+^ cells, CD45^+^ CD34^+^ collagen I^+^ CXCR4^+^ fibrocytes, and HSP47^+^ activated fibroblasts that were accompanied by upregulation of collagen type Iα1, collagen type IIIα1 and NF-kB in lacrimal glands. Collectively, these pre-clinical data help prove the concept that subcutaneous and subconjunctival injection of HC-HA/PTX3 is a novel approach to prevent dry eye disease caused by cGVHD and allow us to test its safety and efficacy in future human clinical trials.

Allogeneic hematopoietic stem cell transplantation (HSCT) is a potentially curative treatment for hematological malignancies. However, chronic graft-versus-host disease (cGVHD) remains a major complication as it may lead to dry eye disease in nearly 40–60% of allogeneic HSCT recipients^1^,^2^,^3^,^4^,^5^,^6^. In fact, dry eye disease is a distinctive sign and symptom for the diagnosis of cGVHD^7^,^8^. A previous study showed that patients with cGVHD-related dry eye disease had decreased conjunctival goblet cell density and corneal sensitivity, meibomian gland obstruction^4^,^9^, tear evaporation, conjunctival squamous metaplasia and inflammatory cellular infiltration^10^. Severe dry eye disease can develop with severe ocular surface damage and dysfunctional tear production with decreased reflex tearing^4^. This is correlated with increased inflammatory cell infiltration in the conjunctiva as evidenced by brush cytology^10^ and biopsy^11^,^12^,^13^ and a high degree of scarring as judged by excessive extracellular matrix accumulation in conjunctiva and lacrimal gland^14^,^15^,^16^. Collectively, these pathological hallmarks of chronic inflammation and scarring highlight the cGVHD-affected lacrimal gland and ocular surface^14^,^17^,^18^.

Several treatments have been used to alleviate dry eye disease associated with cGVHD including topical application of lubricants, retinoic acid, autologous serum, punctual occlusion, moist chambers, tarsorrhaphy, and systemic immunosuppressive treatment with FK-506 and corticosteroids^2^,^17^,^19^,^20^. Nonetheless, none of them has been effective in preventing the development of dry eye disease caused by cGVHD. Cryopreserved human amniotic membrane (AM) has been applied to the ocular surface to reduce inflammation, scarring, and angiogenesis^21^,^22^,^23^. For inflammation, cryopreserved AM has been demonstrated to induce apoptosis of inflammatory neutrophils^24^,^25^, monocytes and macrophages^26^, and to reduce infiltration of inflammatory neutrophils^24^,^25^, macrophages^27^,^28^ and lymphocytes^29^. From human AM, we have purified HC-HA/PTX3 complex that is formed by a covalent linkage between hyaluronan (HA) and the heavy chain 1 (HC1) of inter-α-trypsin inhibitor (IαI)^30^,^31^ and pentraxin 3 (PTX3)^30^. HC-HA/PTX3 as a unique matrix is responsible for the known therapeutic actions of AM’s anti-inflammatory, anti-scarring and anti-angiogenic therapeutic actions^30^,^32^,^33^. Recently, subconjunctival injection of HC-HA/PTX3 has been shown effective in attenuating corneal allograft rejection in a murine model, in which macrophages and CD4^+^ lymphocytes play an important role^30^,^32^,^33^. Herein, we report our continuing endeavors in exploring the therapeutic potential of HC-HA/PTX3 in suppressing inflammatory cellular infiltration and fibrosis in lacrimal glands and conjunctiva of the murine model of cGVHD.

## Results

### HC-HA/PTX3 preserves tear secretion and conjunctival goblet cells

**Figure 1:**
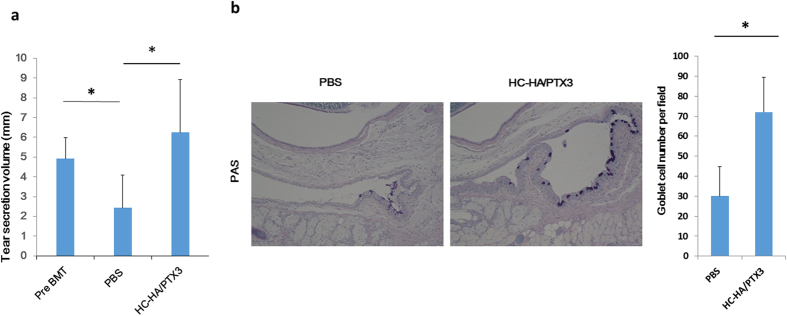
HC-HA/PTX3 maintains tear secretion and conjunctival goblet cells. The average tear volume in mm measured at Day 32 after BMT (i.e., 4 days after the last injection of Post BMT/PBS or Post BMT/HC-HA/PTX3) with a cotton thread. A comparison between the Post BMT/HC-HA/PTX3- and Post BMT/PBS-injected mice was made. In this comparison, the average tear volume measured before BMT was the control (**a)**, *p = 0.023; pre BMT vs post BMT/PBS, *p = 0.034 for post BMT/PBS vs post BMT/HC-HA/PTX3. N = 4 for PBS and N = 6 for HC/HA-PTX3). Conjunctival tissues harvested at Day 32 after BMT, i.e., 4 days after the last injection of PBS or HC-HA/PTX3 were sectioned for PAS staining (**b)**, Scale bar = 100 μm). The number of goblet cells detected by PAS per field was compared between HC-HA/PTX3 - and the PBS –injected conjunctiva (**b)**, *p = 0.012, N = 4 for PBS and N = 5 for HC-HA/PTX3).

Dry eye manifested as low tear secretion and a loss of conjunctival goblet cells is a major complication of cGVHD following allogeneic HSCT. As expected, the average tear secretion volume measured by the cotton thread test was significantly reduced after BMT when compared to the baseline prior to the BMT in PBS-injected controls (*p* = 0.023) (Fig. 1a). In contrast, the average tear volume in mice treated with HC-HA/PTX3 was comparable to that in the pre-BMT normal control (*p* = 0.240), but significantly higher than that in the PBS-treated mice (*p* = 0.034) (Fig. 1a). The conjunctival goblet cell density measured by periodic acid schiff staining showed that HC-HA/PTX3-injected mice had a significantly higher number of conjunctival goblet cells per fields than the PBS-injected ones (*p* = 0.012) (Fig. 1b). Collectively, these results suggest that HC-HA/PTX3 is effective in preserving the normal tear secretion and the normal conjunctival goblet cell density in mice receiving BMT, which otherwise developed cGVHD.

### HC-HA/PTX3 suppresses the infiltration of inflammatory/immune cells in lacrimal glands

**Figure 2:**
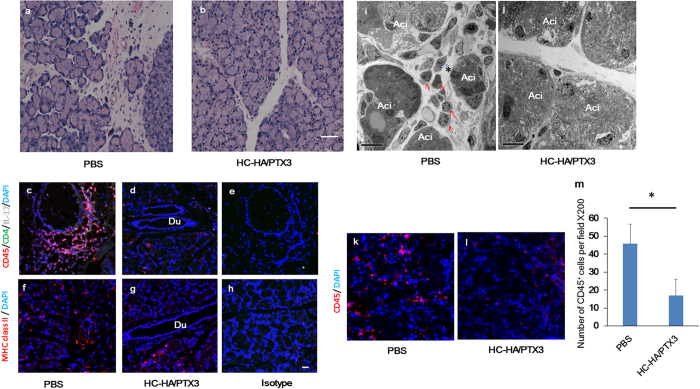
HC-HA/PTX3 reduces infiltration of inflammatory and immune cells into lacrimal glands of cGVHD mice. Lacrimal glands collected at Day 32 after BMT, i.e., 4 days after the last injection of PBS or HC-HA/PTX3, were sectioned for H & E staining (**a**,**b)**, scale bar = 50 μm), immunofluorescence staining against CD45 (Red), CD4 (Green), IL-17 (White) (**c**,**d**), MHC class II (Red) (**f**,**g**), and DAPI nuclear staining (Blue) (**c**–**h)**, scale bar = 20 μm), and transmission electron microscopy (**i** and **j)**, Ac: acinus; red arrows: infiltrating inflammatory cells; asterisks: capillary; scale bar = 10 μm). Immunostaining for CD45 (red) and cell nuclei (blue) (**k** and **l)**, scale bar = 20 μm). The number of CD45^+^ cells per field at x200 magnification in the HC-HA/PTX3- and PBS-treated groups were counted at least 5 different field on 4 sections (**m**), **p = 0.015, N = 4 for each group).

In this proof-of-concept study, we confirmed the reproducibility of these observations by examining the action mechanism of the aforementioned benefit and conducting immunohistochemical and electron microscopic analyses of the murine lacrimal glands. The result showed that a number of mononuclear inflammatory cells infiltrated into the interlobular area of the lacrimal gland in the PBS-injected control group (Fig. 2a) but not in the HC-HA/PTX3-injected group (Fig. 2b). Immunostaining showed that CD45^+^ CD4^+^ IL-17^+^ cells migrated to the interlobular area around lacrimal acini in the PBS group (Fig. 2c), but was greatly subdued in the HC-HA/PTX3 group (Fig. 2d). Isotype matched control showed no staining (Fig. 2e). The majority of CD45^+^ inflammatory cells were CD4^+^ Th 17 cells, in addition to CD8^+^ cells, CD11c^+^ cells, CD19^+^ cells, and CD68^+^ cells in the cGVHD lacrimal gland in the PBS group. In contrast, infiltration of these cells was also suppressed by the HC-HA/PTX3 injection (data not shown). Furthermore, MHC class II^+^ cells migrated to the interlobular area in the PBS group (Fig. 2f), but were markedly decreased in the HC-HA/PTX3 group (Fig. 2g). The isotype-matched control showed no staining (Fig. 2h). Transmission electron microscopy and immunofluoresence images also revealed a variety of mononuclear cells migrated in the lobules of lacrimal gland in the PBS-treated mice (Fig. 2c,i,k). In contrast, such cellular infiltration was reduced in the HC-HA/PTX3 group (Fig. 2d,j,l). Quantitative analysis showed that the number of CD45^+^ cells per field in the HC-HA/PTX3 group was significantly lower than that in the PBS group (p = 0.015) (Fig. 2m). Collectively, these results suggest that HC-HA/PTX3 suppresses cGVHD-triggered inflammatory and immune cellular infiltration in the lacrimal gland.

### HC-HA/PTX3 attenuates cGVHD-induced interstitial fibrosis

**Figure 3:**
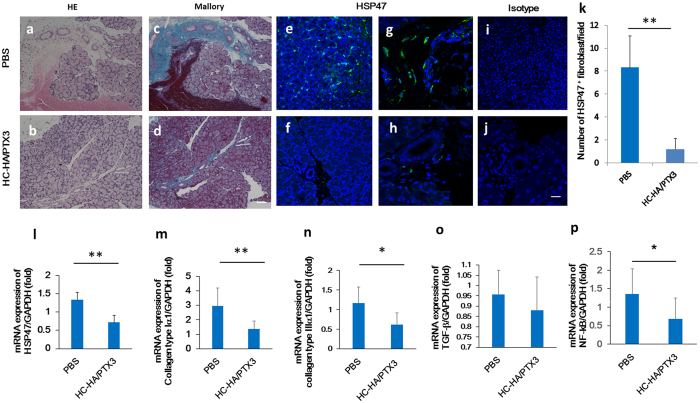
HC-HA/PTX3 inhibits interstitial fibrosis in lacrimal gland of cGVHD mice. Lacrimal glands harvested at Day 32 after BMT, i.e., 4 days after the last injection of PBS or HC-HA/PTX3, were sectioned for H & E (**a**,**b**), Mallory (**c**,**d**) staining (Scale bar = 100 μm), immunofluorescence staining of HSP47 (green) (**e**,**i** and **f)**, Scale bar = 50 μm, G, H, and J, Scale bar = 20 μm), quantitative comparison of the number of HSP47^+^ fibroblasts per field (**k)**, **p = 0.006 for HSP47, N = 8 for PBS and N = 12 for HC-HA/PTX3). Transcript levels of each molecules, *p* = 0.006 for HSP47(L), *p* = 0.004 for collagen type Iα1 (**m**), *p = 0.033 for collagen type IIIα1 (**n**), not statistically significant for TGF-β1 (**o**) *p = 0.038 for NF-kB (**p**) by quantitative real-time PCR analyses using the expression of GAPDH as the internal control (*p < 0.05, **p < *0.01,* N = 5 for PBS and N = 12 for HC-HA/PTX3). The HSP47^+^ cells per field (at x 400 magnification) were counted in at least 5 different fields on 3–5 sections.

Consistent with our previous report about immune-mediated fibrosis in lacrimal glands with cGVHD^5^, H&E staining and Mallory staining of the lacrimal gland demonstrated that excessive fibrosis was observed around medium sized ducts in the PBS-treated mice (Fig. 3a and c). In contrast, this pathological change was substantially repressed in the HC-HA/PTX3-treated mice (Fig. 3b and d). Further characterization by immunostaining showed that HSP47^+^ activated fibroblasts was readily seen in the lacrimal gland of the PBS injection group (Fig. 3e and g), but markedly diminished in the HC-HA/PTX3 group (Fig. 3f and h). The isotype-matched control showed no staining (Fig. 3i and j). Quantitative analysis confirmed that the number of HSP47^+^ cells/field was significantly decreased in the HC-HA/PTX3 group when compared to that in the PBS group (*p* = 0.006) (Fig. 3k). In addition, the transcript expression of HSP47 (*p* = 0.006), collagen type Iα1 (*p* = 0.004), and collagen type IIIα1 (*p* = 0.033) was significantly declined in the HC-HA/PTX3 group (Fig. 3l,m,n, respectively). Although the transcript expression of TGF-β in the HC-HA/PTX3 group was lower than that in the PBS group, such a difference did not reach a statistical significance (Fig. 3o). The transcript level of NF-κB, a critical T cell-related inflammatory transcription factor, was significantly decreased in the HC-HA/PTX3 group when compared to that in the PBS group (*p* = 0.038) (Fig. 3p). Taken together, these results suggest that HC-HA/PTX3 can also effectively suppress fibroblast activation and curtail the expression of fibrosis-related genes, thus mitigating fibrosis in the lacrimal gland caused by cGVHD.

### HC-HA/PTX3 suppresses infiltration of inflammatory cells and fibrocytes

**Figure 4:**
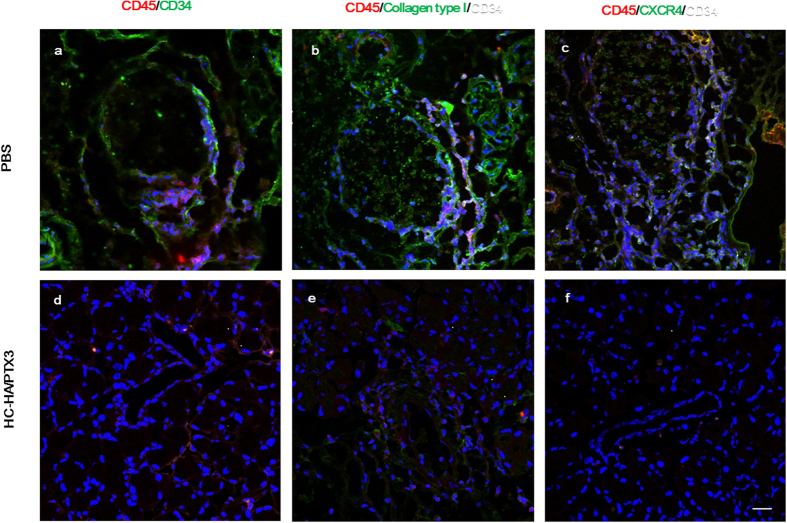
HC-HA/PTX3 suppresses inflammatory cell and fibrocyte infiltration into lacrimal glands of cGVHD mice. Lacrimal glands collected at Day 32 after BMT, i.e., 4 days after the last injection of PBS or HC-HA/PTX3 were sectioned for immunostaining of CD45 (red)/CD34 (green) (**a**,**d**), CD45 (red)/collagen type I (green) and CD34 white (**b**,**e**), and CD45 (red)/CXCR4 (green) and CD34 (white) (**c**,**f**). Scale bar = 20 μm.

Previously, we reported that a subpopulation of fibroblasts could contribute to fibrosis in human lacrimal gland disordered by cGVHD^5^. Our subsequent study indicated that these fibroblasts were derived from fibrocytes, the generation of which was presumably elicited by cGVHD^5^. To detect fibrocytes in the lacrimal gland, we performed triple staining for CD45^+^, CD34^+^ and CXCR4^+^ cells and for CD45^+^, CD34^+^ and collagen type I^+^ cells. As expected, some CD45^+^ cells co-expressing CD34, CXCR4 and collagen type I were detected around blood vessels and ducts in the lacrimal gland of the PBS-injected group (Fig. 4a,b and c, respectively), confirming the involvement of fibrocytes in fibrosis of the lacrimal gland in cGVHD. In contrast, the number of fibrocytes was significantly reduced in the HC-HA/PTX3 group (Fig. 4d,e,f). These results further suggest that fibrocytes contribute to the inflammation and excessive fibrosis in the lacrimal gland of affected by cGVHD and that HC-HA/PTX3 can reduce the cGVHD-elicited inflammation and fibrosis by subduing the migration of these fibrocytes.

### HC-HA/PTX3 suppresses inflammation and fibrosis in conjunctiva

**Figure 5:**
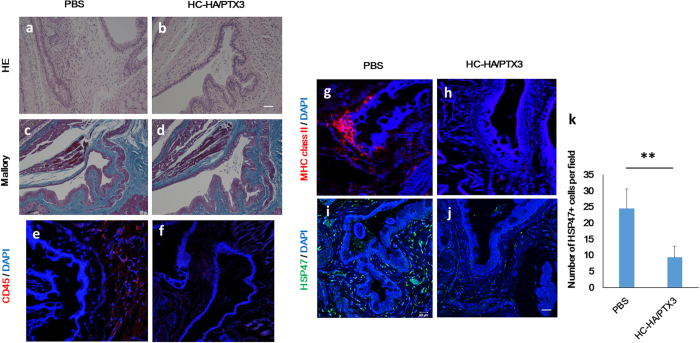
HC-HA/PTX3 suppresses inflammation and fibrosis in conjunctiva of cGVHD mice. Conjunctiva harvested at Day 32 after BMT, i.e., 4 days after the last injection of PBS or HC-HA/PTX3, were sectioned for H & E (**a** and **b)**, Scale bar = 100 μm) and Mallory staining (**c** and **d**), Scale bar = 100 μm) and immunostaining for CD45^+^ cells (red) (**e** and **f**), MHC class II^+^ cells (red) (**g** and **h**) and HSP47^+^ activated fibroblasts (green) (**i** and **j**) for both groups (Scale bar = 50 μm for (**e**–**h**). The number of HSP47^+^ cells/field was compared between the two groups (**p = 0.005, N = 4 for PBS and N = 4 for HC-HA/PTX3).

We then performed morphological analyses using H & E staining and Mallory staining to determine whether inflammation and fibrosis, respectively, was also affected in the conjunctiva by HC-HA/PTX3. The result showed infiltration of mononuclear cells (Fig. 5a) and dense fibrotic area (Fig. 5c) in the PBS group but not in the HC-HA/PTX3 group (Fig. 5b and d). Immunostaining confirmed infiltration of CD45^+^ cells (Fig. 5e) and MHC class II^+^ cells (Fig. 5g) in the conjunctiva of the PBS group, whereas such cellular infiltration was suppressed in the HC-HA/PTX3 group (Fig. 5f and h). Furthermore, HSP47^+^ activated fibroblasts were found in the conjunctiva of the PBS group (Fig. 5i) but were markedly diminished in that of the HC-HA/PTX3 group (Fig. 5j). The isotype-matched control showed no staining (data not shown). Quantitative analysis confirmed that the number of HSP47^+^ cells/field was significantly decreased in the HC-HA/PTX3 group when compared to the PBS group (*p* = 0.005) (Fig. 5k). These results suggest that both inflammation and fibrosis in the conjunctiva were also suppressed by HC-HA/PTX3.

## Discussion

Dry eye disease is a major complication after HSCT in humans. Similar to human cGVHD, reduced tear secretion and goblet cell density were seen in the PBS-injected control group of our murine cGVHD model. Nonetheless, these two pathological hallmarks of dry eye disease were not observed in the same murine cGVHD model receiving subconjunctival and subcutaneous injection of HC-HA/PTX3 twice a week from Day 4 to Day 28 after BMT. Hence, we conclude that HC-HA/PTX3 purified from human AM could have a potential to treat cGVHD-related dry eye disease.

Our data also suggest that the aforementioned therapeutic benefit of HC-HA/PTX3 is directed to suppressing infiltration of inflammatory/immune cells to the lacrimal gland triggered by cGVHD. In human cGVHD, T cells play a critical role for pathogenic process of inflammatory cascades as a plenty of HLA-DR^+^ cells infiltrate into lacrimal glands. The infiltration of CD4^+^ Th 17 effector cells as well as CD45^+^ and MHC class II^+^ cells around lacrimal gland ducts was significantly reduced by HC-HA/PTX3 (Fig. 2a–j). These results imply that HC-HA/PTX3 could inhibit the effector phase of T cell activation, a notion that was also suggested by its suppression of NF-κB in the lacrimal gland tissue (Fig. 3p). Collectively, these findings are also consistent with our previous findings that HC-HA/PTX3 inhibits M1 macrophages infiltrating onto LPS-elicited mouse corneas and further polarizes these infiltrated macrophages towards M2 phenotype^32^ and that subconjunctival injection of HC-HA/PTX3 prolongs the survival of allogeneic corneal grafts in a murine model^32^. In mice, the infusion of IL-23-deficient splenocytes, or the use of specific IL-23 antibody decreases GVHD associated morbidity^34^. Neutralization of IL-12 can also be an effective way of preventing acute GVHD^34^. Because HC-HA/PTX3 can downregulate expression of both IL-12 and IL-23 in IFN-γ/LPS activated macrophages^30^, these potential modes of action bode well for considering HC-HA/PTX3 as a novel agent to control inflammation mediated by inflammatory and immune cells in cGVHD.

Besides inflammation, fibrosis is another prominent pathological hallmark of cGVHD^5^,^17^. By interacting with T cells, fibroblasts originating from circulating donor-derived precursors and recipient derived fibroblasts can play a role in the excessive fibrosis in patients with cGVHD^15^,^16^. Indeed, we have reported that CD34^+^ fibroblasts are increased at the sites of acinar cell loss and in the fibrotic areas around the ducts in patients with cGVHD^16^. CD34 is initially known as a marker of hematopoietic stem cells, but later it was reported also expressed in endothelial cells and some of dendritic cells and interstitial fibroblasts in normal submandibular glands^35^. Another source of CD34^+^ fibroblasts in the lacrimal gland might be donor-derived fibrocytes, a population of blood-borne cells with a spindle-shaped morphology and distinctive cell-surface markers (collagen I^+^/CXCR4^+^/CD34^+^/CD45^+^)^36^,^37^. Herein, we confirmed an increasing number of HSP47^+^ activated fibroblasts that were accompanied by upregulation of collagen type Iα1 and collagen type IIIα1 transcripts (Fig. 3) as well as donor- or recipient-derived fibrocytes which were CD45^+^ cells co-expressing CD34, CXCR4 and collagen type I (Fig. 4) in lacrimal glands of the PBS group. Those cells are presumably donor-derived fibrocytes, but there is a possibility that host-derived cells which survived radiation are also the source of fibrocytes. Importantly, such pathological features were also suppressed by HC-HA/PTX3 injection. Suppression of inflammation and fibrosis by HC-HA/PTX3 was also effectively extended to the conjunctiva (Fig. 5). Collectively, although this additional therapeutic benefit in preventing fibrosis can be explained by the anti-inflammatory action of HC-HA/PTX3 discussed above, we cannot ignore the fact that HC-HA/PTX3 can also exert a direct effect on downregulation of TGF-β expression as reported in corneal fibroblast^38^,^39^. This notion is further supported by the finding that AM and its active component HC-HA/PTX3 can prevent phosphorylation and nuclear translocation of Smad2/3 and thereby inhibit transcription of the TGF-β family and TGF-β receptors^38^, the production of TGF-β1 and TGF-βRII (unpublished data), and the signal transduction of TGF-β1^40^.

Collectively, these potential therapeutic benefit exerted by HC-HA/PTX3 against inflammation and fibrosis mediated by T-cell activation and infiltration of donor-derived fibrocytes is ideal to mitigate dry eye disease caused by cGVHD. Our study described herein may open a new avenue for a novel therapeutic intervention for this devastating disease. Hence, this pre-clinical study justifies our further investigation of HC-HA/PTX3 as a new biologic for treating human cGVHD-related dry eye disease patients.

## Materials and Methods

All animal experiments were conducted in accordance with the ARVO Statement for the Use of Animals in Ophthalmic and Vision Research and the protocol #09152 approved by the Ethics Committee on Animal Research of the Keio University School of Medicine. We followed the ARRIVE guidelines for reporting *in vivo* experiments in animal research^41^.

### Materials

All chemicals for preparation of HC-HA/PTX3 from human AM were from Sigma-Aldrich (St. Louis, MO). HA quantitation kit was from Corgenix (Broomfield, CO). BCA Protein Assay Kit was from Pierce (Rockford, IL). The 4–15% gradient acrylamide ready gels and nitrocellulose membranes was from BIO-RAD (Hercules, CA). HC1 antibody (ab70048) was from abcam (Cambridge, MA) and PTX3 antibody (MNB1, ALX-804-463-C100) was from Enzo Life Sciences, Inc. (Farmingdale, NY). Antibodies against CD3 (17-0031-63, Clone 145-2C11) and MHC class II (12–5321, Clone M5/114.15.2) were from eBioscience (San Diego, CA). Antibodies against CD4 (17-0041-81, Clone GK1.5), CD8 (14-0081-81, Clone 53–6.7), CD11c (14-0114-81, Clone N418), CD19 (14-0193-81, Clone eBio1D3 (1D3), CD34 (ab8158, Clone MEC 14.7), and CD 45 (12-0451-81, Clone 30-F11) were from BD Pharmingen (San Jose, CA). CD68 antibody (MCA 1957GA, Clone FA-11) was from DS Pharma Biomedical (Osaka, Japan) and CD154 antibody (106502, Clone MCA 1957GA) was from BioLegend (San Diego, CA). IL-17 antibody (SC-6076, Clone C-20) was from Santa Cruz Biotechnology (Dallas, Texas). Heat shock protein 47 (HSP47) antibody (SPA-470) was from Stressgen Biotechnologies Corp. (San Diego, CA). CXCR4 antibody (ab2074) were from abcam (Cambridge, MA). Isotype matched antibodies used as negative controls were as follow: (1) mouse Ig G 2b, κ antibody for HSP47; (2) rat Ig G2b, κ antibody for CD4, CD45, and MHC class II; (3) rat IgG2a antibody for CD68; (4) rat IgG2a, κ antibody for CD8, CD34, and IL-17; and (5) Armenian hamster IgG antibody for CD3, CD11c, CD19, CD154, and CXCR4. Cell nuclei were stained with 4’, 6-Diamidino-2-Phenylindole (DAPI) (Thermo Fisher Sientific, Tokyo, Japan). RPMI 1640 was purchased from BioWhittaker (Walkersville, MD). The antigen retrieval solution (Histo VT One) and Target Retrieval Solution were from Nacalai Tesque (Tokyo, Japan) and Dako (Glostrup, Denmark), respectively.

A RNeasy mini kit and a ReverTra Ace qPCR RT Kit with genomic remover were from Qiagen (Valencia, CA). The primers (for glyceraldehyde 3-phosphate dehydrogenase or GAPDH, HSP47, collagen type Iα1, collagen type IIIα1, TGF-β and NF-kB) for mRNA expression analysis by TaqMan real-time PCR were from Applied Biosystems (Applied Systems Inc, Ontario, Canada).

### Preparation of HC-HA/PTX3 from human AM

As reported^42^, HC-HA/PTX3 was prepared from frozen human placentas provided by Bio-Tissue, Inc. (Miami, FL). After separation from other placental tissues (e.g., chorion) under aseptic conditions, AM was sliced into small pieces and frozen at −80 °C or briefly in liquid nitrogen, followed by homogenization in a Cuisinart CBT-700 blender (East Windsor, NJ) at a ratio of 1:1 [PBS volume (ml)/AM weight (g)]. The homogenate was mixed at 4 °C for 1 h and the supernatant, termed AM extract (AME), was collected after centrifugation at 48,000 g at 4 °C for 30 min. AME was further subjected to two runs of ultracentrifugation at 35,000 rpm (the maximum g force at 245, 853 × g, Optima L-80XP, SW 41, Indianapolis, IN) in CsCl/4 M guanidine HCl at an initial density of 1.35 g/ml (the first run) and 1.40 g/ml (the second run) for 48 h at 15 °C. Fractions containing HA (measured by HA Quantitative Test Kit) but no detectable amount of proteins (measured by BCA assay) were pooled, dialyzed extensively against distilled water, and designated as HC-HA/PTX3. Therefore, the amount of HC-HA/PTX3 was expressed based on the HA amount present in the complex.

### Bone marrow transplantation (BMT) to produce a mouse model of cGVHD

Two 8-week-old male B10.D2 (H-2d) and ten 8-week-old female BALB/c (H-2d) mice were obtained from Sankyo Laboratory, Ltd (Tokyo, Japan). Allogeneic BMT was conducted as previously reported^43^ using the B10.D2 and BALB/c mice as transplant donors and recipients, respectively. Prior to BMT, the female recipient mice were lethally irradiated with 700 cGy from a Gammacel 137Cs source (J. L. Shepherd & Associates, San Fernando, CA). Approximately 6 h after the irradiation, each of the recipient mice was injected with 1 × 10^6^ male donor bone marrow and 2 × 10^6^ spleen cells. The donor cells were suspended in RPMI 1640, and the transplantation was performed by tail vein injection. BMT to produce this animal model of GVHD can be usually conducted with a success rate of approximately 90%^43^,^44^,^45^. The experiment was repeated three times to ensure its reproducibility.

### Subconjunctival and subcutaneous injection of HC-HA/PTX3

Ten allogeneic BMT recipient BALB/c mice were divided into two groups: HC-HA/PTX-treated group (n = 5) and the solvent PBS-treated group (n = 5). Each time the mice were medicated with HC-HA/PTX3 or PBS, they underwent two subconjunctival injections and two subcutaneous injections. The administration procedures were as follows: (1) 10 μl of HC-HA/PTX3 (1 mg/ml) or PBS was injected at each injection site and (2) the injections were carried out using a 30G needle. The concentration of HC-HA/PTX3 and the amount of solvent were determined based on our previous report^32^. Such injections were performed twice a week, every 3 or 4 days, from Day 4 to Day 28 after BMT. Each mouse was treated with HC-HA/PTX3 or PBS 7 times in total.

### Cotton thread test for measuring lacrimal tear secretion

A phenol red thread was placed on the temporal side of the lower eyelid margin for 15 seconds. The length of the moistened thread from the edge was measured in both eyes and the average was used as lacrimal tear volume for comparative analysis^43^.

### Histochemical and Mallory staining

Formalin fixed, paraffin-embedded tissue sections were processed using conventional histological techniques, including hematoxylin and eosin (H & E) staining and Mallory staining^46^,^47^. Tissue sections of lacrimal gland and conjunctiva subjected to Mallory staining were assessed for morphometric analysis. A minimum of four randomly selected fields were captured at 200× magnification for each section using a Nikon Coolscope II (Nikon Corp., Tokyo, Japan) and imported into a computerized image analysis system-Image J (NIH, Bethesda, MD). The degree of fibrosis 35 days after BMT was analyzed. For lacrimal gland tissue, the collagen deposition was quantified as the ratio of the blue-stained area to the total stained area and expressed as % fibrotic area^43^,^48^. To assess the histological architecture and staining, each slide was reviewed twice independently by each of two observers, who were blinded to which group the specimen was from in the morphometric observation.

### Immunostaining

Immunohistochemistry was performed on formalin-fixed paraffin-embedded or frozen tissue sections. The staining patterns were graded semi-quantitatively according to the intensity and distribution of the labeling, as described previously^14^. For immunofluorescent staining of HSP47, a collagen-specific molecular chaperon, antigen unmasking was done for paraffin-embedded sections by autoclaving at 120 °C for 20 min or incubating the sections at 37 °C in antigen retrieval solution (Histo VT One, Nacalai Tesque, Tokyo, Japan) for 10 min for formalin fixed-frozen sections. For cell-surface immunostaining of CD45, paraffin-embedded sections were microwaved for 10 min in 10 mM sodium citrate buffer, pH 6.0. The sections were then blocked with 10% goat serum for 1 h and incubated overnight at 4 °C with an anti-CD3, CD4, CD8, CD11c, CD19, CD45, CD68, CD154, IL-17, MHC class II and HSP47 antibody. After washing with PBS, the sections were incubated with an Alexa 488-conjugated goat anti-mouse, rabbit or hamster secondary antibody (Molecular Probes, Grand Island, NY) and the nuclei were stained simultaneously with DAPI. Tissue sections for fluorescent staining were examined with an LSM 700 confocal microscope (Carl Zeiss, Jena, Germany). To assess the histological architecture and staining, all of the acquired images were reviewed twice independently by each of two observers who were blinded to the source of the samples. The number of HSP47^+^ fibroblasts/field (at x400 magnification) and CD45^+^ cells per field (at x200 magnification) were counted in at least 5 different fields on 3 to 5 sections. HSP47^+^ spindle-shaped cells with oval nuclei that resided in the interstitium were regarded as fibroblasts^16^. The positive immunostaining of HSP47, a collagen specific molecular chaperon, was used as a marker of activated fibroblasts.

### Transmission electron microscopy

A portion of lacrimal gland and conjunctival tissues was immediately fixed with 2.5% glutaraldehyde and subjected to electron microscopic examination as described previously^49^. One-micrometer-thick sections were stained with methylene blue, and the portions of interest were thin-sectioned and examined under an electron microscope (1230 EXII; JOEL, Tokyo, Japan). All photographs were taken with a bio scan camera (Gatan bio scan camera model 792, Tokyo, Japan).

### Quantitative real-time polymerase chain reaction (qPCR)

Total RNA was extracted from lacrimal glands using a RNeasy mini kit, and cDNA synthesis was performed using a ReverTra Ace with genomic remover qPCR RT Kit. Quantitative real time PCR was performed using the Step One Plus system (Applied Biosystems, Grand Island, NY). All data were analyzed with the 2 ΔΔCT method, and the mRNA of GAPDH was used as the internal standard. We used the following mouse TaqMan probe for quantitative real time PCR: Mm00438058_g1 for HSP47, Mm00801666_g1 for collagen type Iα1, Mm01254476_m1 for collagen type IIIα1, Mm01178820_m1 for TGF-β 1 and Mm00479809_g1 for NF-kB, and Mm99999915_g1 for GAPDH.

### Statistical Analysis

The unpaired Student’s t-test or nonparametric one-way analysis of variance (ANOVA) was used to test for differences among groups. Experiments were repeated for two times. Summary data were reported as means ± S.D. A calculated probability (p) value less than 0.05 (p < 0.05) was considered statistically significant.

## Additional Information

**How to cite this article:** Ogawa, Y. *et al*. Heavy Chain-Hyaluronan/Pentraxin 3 from Amniotic Membrane Suppresses Inflammation and Scarring in Murine Lacrimal Gland and Conjunctiva of Chronic Graft-versus-Host Disease. *Sci. Rep.* **7**, 42195; doi: 10.1038/srep42195 (2017).

**Publisher's note:** Springer Nature remains neutral with regard to jurisdictional claims in published maps and institutional affiliations.
